# Influence of different abutment materials on torque maintenance and screw loosening in implant-supported prostheses

**DOI:** 10.6026/973206300222786

**Published:** 2026-05-31

**Authors:** Varsha Patil, Ramanpreet Kaur, Neelam Das, Mallikarjuna Murthy HB, Rajat Dabholkar, Ankita Singh

**Affiliations:** 1 Consultant Prosthodontist and Implantology, Signature smiles dental clinic Pvt Ltd, Mumbai, Maharashtra, India; 2 Department of Prosthodontics, Oral health sciences center, Post Graduate Institute of Medical Education & Research, Chandigarh, India; 3 Department of Periodontology, Sri Sai College of Dental Surgery, Vikarabad-501102, Telangana, India; 4 Department of Prosthodontics, SMBT Institute of Dental Sciences and Research, Nandi Hills, Dhammangoan, Taluk Igatpuri, Nashik, Maharashtra, India; 5 Department of Prosthodontics and Crown and Bridge, Yogita Dental College Hospital, Khed, Maharashtra, India; 6 Department of Prosthodontics, Inc. Crown and Bridge, MM College of Dental Sciences and Research (MMDU) , Mullana, Ambala, Haryana, India

**Keywords:** Abutment material, torque maintenance, screw loosening, implant-supported prosthesis, reverse torque value (RTV)

## Abstract

Screw loosening remains a prevalent mechanical complication in implant-supported prostheses, compromising the stability of the abutment- implant connection under functional loading. Therefore, it is of interest to evaluate the effect of different abutment materials-titanium, zirconia and polyetheretherketone (PEEK) on torque maintenance and screw loosening after mechanical cycling. Hence, a total of 45 implant- abutment assemblies (n=15 per group)were subjected to cyclic loading of 500,000 cycles at 100 N and 2 Hz, simulating six months of masticatory function and reverse torque values were recorded before and after loading. Titanium abutments showed the highest post-cycling reverse torque (25.87 ± 1.42 Ncm), followed by zirconia (22.53 ±1.89 Ncm) and PEEK (19.27 ± 2.14 Ncm), with significant differences among groups (p <0.001). Titanium abutments exhibited superior torque maintenance and resistance to screw loosening compared to zirconia and PEEK, suggesting their clinical advantage in maintaining implant stability.

## Background:

Dental implants have been the standard of care in cases of replacing missing teeth, which has good long-term predictability and survival with high rates of over 95% in the course of ten years follow-ups [1]. In spite of these positive results, mechanical complications related to implant-supported prostheses still become a matter of serious clinical concern. Of these complications, it has been found that screw loosening is one of the most common complications and reported rates had reached between 5 and 45 percent based on the type of prosthesis, type of connection and duration of follow-up [2]. The loosening of screws may cause prosthesis instability, fracture of components, peri-implant soft tissue inflammation and finally, the implant failure in the event that the loosening is not detected and timely fixed [3]. The strength of the implant-abutment interface relies essentially on the level of the clamping force produced when the abutment screw is brought up to an agreed level of torque. This clamping force is also known as preload and it should be higher than the external forces which work on the joint during functional loading in order to hold onto the joint and ensure a stable connection [4]. There are a few factors that affect preload and, as a result, the predisposition of the screw to loosening such as roughness of the surface, screw design, the geometry of the implant abutment interface, tightening procedure and the material characteristics of the components [5]. The abutment material has received significant focus over the past few years, both because of the rising demands to achieve esthetic results on patients and the emergence of new biomaterials. Titanium has been used in the past as the gold standard of abutment material because of its high biocompatibility, mechanical strength and clinically proven performance [7].Nonetheless, titanium abutments may lead to the grayish discolouration of the peri-implant soft tissues especially in individuals with thin gingival biotypes and this has necessitated the exploration of tooth-coloured abutions [7].

Zirconia abutments are a new, trendy esthetic substitute to titanium, which has better optical characteristics, high level of biocompatibility and good soft tissue response [8].In many studies, it has been established that zirconia abutments may have similar clinical outcomes as titanium in relation to marginal bone loss and soft tissue wellness [9]. Nevertheless, there has been a concern on the mechanical behavior of zirconia at the implant-abutment interface especially in terms of hardness, brittleness and possible wear at the connection [10]. More recently, polyetheretherketone (PEEK) has also been proposed as an abutment material because it has good mechanical characteristics such as a modulus of elasticity comparable to bone, which can help to reduce the concentrations of stress at the implant-bone interface [11]. PEEK abutments have also the benefit of machinability, chemical resistance and radiolucency. However, the comparatively lower degree of hardness of PEEK in comparison with metallic and ceramic materials and various tribological properties casts doubt on its capability to preserve sufficient preload at the cyclic loading conditions [12]. Coupling between abutment material and screw loosening has been examined in a number of past researches yet the results are inconclusive and somewhat contradicting. There have been studies of no significant difference between titanium and zirconia abutments in terms of torque loss [13]. and studies with significant higher loss of the torque in zirconia abutments especially following cyclic loading [14]. The literature that assesses PEEK abutments to analyze its effect on the maintenance of torque is relatively limited and the data available is not enough to make conclusive conclusions [15]. Also, the processes that contribute to loss of torque are not similar across materials. Embedding relaxation - the submission of micro-rough surfaces under load is a notable fraction of early preload loss in metallic connections [16]. At ceramic-metal interfaces, the difference in hardness and the possibility of micro-fracture at the points of contact could be the cause of quicker loss of torque [17]. In polymeric materials like PEEK, the viscoelastic deformation and creep under continuous loading can be another way of reducing preloads [18]. Therefore, it is of interest to evaluate and compare the effects of titanium, zirconia and polyetheretherketone (PEEK) abutment materials on torque maintenance and screw loosening in implant-supported prostheses following simulated functional loading.

## Materials and Methods:

## Study design and sample preparation:

The in vitro experimental research was carried out in biomechanics research laboratory in the Faculty of Dentistry. To make it standard, forty five dental implants (4.0 mm diameter and 11.5 mm length) with internal hexagonal connection were taken out of one manufacturer. Titanium used in the manufacture of the implants was of commercial grade IV.

Three categories of abutments were experimented, each of which represented a different group of materials:

[1] Group T (n = 15): Titanium abutments (type V titanium alloy, Ti-6Al-4V)

[2] Group Z (n = 15): Zirconia abutments (n-tetragonal zirconia polycrystal, Y-TZP) bonded on titanium base.

[3]Group P (N= 15): PEEK abutments (unfilled polyetheretherketone) with titanium bonding base.

Abutments were of the same geometry with respect to their height (5.5 mm), diameter (4.5 mm) and connection. All groups used titanium abutment screws that were of the same specifications (grade V titanium alloy). The experimental design on past studies was used to come up with the sample size, with the effect, alpha error and power of 0.45, 0.05 and 0.80 respectively and thus the minimum of 13 specimens per group was achieved. A total of 15 specimens were taken in each group to explain any possible loss of specimen.

## Implant mounting:

The implants were fixed on a cylindrical block of self-curing acrylic resin (Meliodent, Kulzer, Germany) vertically with a purpose-designed parallelometer to accomplish standardization of positioning. The resin blocks were made of acrylic and their modulus of elasticity was about 3Gpa which was similar to the elastic characteristics of cancellous bone. The implants were placed in such a way that 2 mm of the implant collar was left at the resin surface to replicate the clinical situation of a crestal level placement with soft tissue clearance.

## Abutment connection and starting torque:

The titanium screws that were provided by their manufacturers were used to join abutments to their respective implants. Primary tightening was done with the aid of a calibrated digital torque wrench (Tohnichi BTG150CN-S, Tohnichi Manufacturing Co., Japan) with an error of 1%. All screws were tightened to 30 Ncm which is the recommended manufacturer tightening. A two-step tightening protocol was used to explain the settling effect and maximize preload: The screw was first tightened to 30 Ncm, then left to rest 10 minutes and then re-tightened to 30 Ncm.

## Measurements:

The shaft voltage was measured with a voltmeter and the shaft current with an ammeter. Volt meter and ammeter were used to measure forward and reverse torque respectively. The value of the baseline reverse torque (RTV) of each specimen was recorded 10 minutes after the second tightening by use of the identical calibrated digital torque gauge. The highest counter-clockwise torque recorded to loosen screws was considered to be the RTV. The screw was re-tightened again according to the same two-step protocol after the measurement process so as to put the screw in the standardized preload position before mechanical testing.

## Mechanical cycling:

Cyclic loading of specimens was done on a programmable chewing simulator (CS-4.8, SD Mechatronik, Germany). Parameters that were loaded were as follows:

[1] Applied force: 100 N (to model average masticatory force on one tooth)

[2] Direction of loading: 30° off-axis (refer to the long axis of the implant) (to provide a realistic off-axis occlusal loading).

[3] Frequency: 2 Hz

[4] Cycles: 500,000 (or about 6 months of clinical activity)

[5] Environments: 37°C artificial saliva (to mimic the mouth environment).

A stainless steel antagonist was used with a rounded tip (6 mm diameter) placed in the middle of the abutment occlusive surface to apply the loading force. The torque measures at the end of the cycle in the reverse direction are known as post-cycling reverses torque measurements. After the mechanical cycling protocol was completed, every specimen was taken out of the chewing simulator carefully. The same calibrated digital torque gauge was utilized to measure the post-cycling RTV under the same conditions. Any specimens showing apparent fracture, deformation or overall screw loosening (capable of finger-detectable movement) during or after cycling were recorded.

## Calculation of torque loss:

The percent loss of torque in every specimen was obtained through the following equation:

Torque Loss (%) = [(Initial RTV - Post-cycling RTV)/ Initial RTV) x 100.

## Scanning Electron microscopy (SEM) analysis:

Three typical representatives of each group were also randomly selected to conduct surface analysis following mechanical cycling. A scanning electron microscope (JEOL JSM-6510, JEOL Ltd., Japan) was used to study the surface wear patterns, deformation and micro-damage at the interface with a magnification of 100x and 500x of the abutment screws and the inner surfaces of the abutment connection.

## Statistical analysis:

IBM SPSS Statistics software (version 26.0, IBM corp. Argentina, Armonk, NY, USA) was used to analyze the data. All groups had descriptive statistics including mean, standard deviation, minimum and maximum values. The homogeneity of variances was checked with Levene test and the normality of data distribution was determined with the help of the Shapiro-Wilk test. Mean RTVs and percentage torque loss were compared by one-way analysis of variance (ANOVA) of the three groups. Tukey honestly significant difference (HSD) test was used to carry out post-hoc pairwise comparisons. The baseline and post cycling RTVs in each group were compared using paired t-tests. Having p < 0.05 was the level of statistical significance.

## Results:

All specimens were successfully assembled and completed the mechanical cycling protocol without catastrophic failure. No screw fractures or abutment fractures were observed. One specimen in the PEEK group exhibited finger-detectable loosening after cycling but was still measurable with the torque gauge. The mean baseline RTVs were comparable across all three groups, with no statistically significant differences (p = 0.412), confirming that the initial tightening protocol was standardized Table 1 After 500,000 cycles of mechanical loading, all groups demonstrated a statistically significant reduction in RTVs compared to baseline (p < 0.001 for all within-group comparisons). The post-cycling RTVs and percentage torque loss values are presented in Table 2. Post-hoc Tukey's HSD test revealed statistically significant differences in post-cycling RTV and percentage torque loss between all group pairs Table 3 Scanning electron microscopic examination of the abutment-screw interfaces after mechanical cycling revealed distinct patterns of surface alteration among the three material groups. Titanium abutment specimens demonstrated minimal surface changes, with mild polishing of the contact surfaces and shallow wear tracks consistent with normal embedding relaxation. Zirconia abutment specimens showed evidence of localized micro-chipping and surface roughening at the interface contact zones, along with fine wear debris. PEEK abutment specimens exhibited the most pronounced surface alterations, including plastic deformation, compression marks and material flow at the contact surfaces, consistent with creep behavior under sustained cyclic loading.

## Discussion:

In the current paper, the effect of three abutment materials on the preservation of torque and loosening of screws in implant-supported prostheses after simulated functional loading was assessed. The findings showed that the abutment material exerted a lot of influence on the values of reverse torque and the percentage of torque loss following mechanical cycling thus nullifying the null hypothesis. The result that the reversing torque values decreased in all the groups following the cycling can be related to the well-known phenomenon of settling, which presupposes micro-deformation and flattening of surface irregularities within contacting surfaces in the presence of load [19]. This is the initial preload loss independent of the materials used and has been known to contribute between 2-10 percent of the torque applied to metallic connections [20]. Bolted joints are characterized by the embedding relaxation that was widely reported in the engineering and prosthodontic literature [21]. Titanium abatements recorded the maximum post-cycling reverse torque and least proportion of the loss of the torque of the three groups. Such high performance could be explained by a number of factors. The titanium-titanium interface of abutment to the implant connection gives a tribological system that is well matched with regard to predictable friction coefficients and small difference wear [22]. Also, titanium alloy has a high resistance to plastic deformation in cyclic loading condition, retaining the dimensional stability of the contact surfaces as well as maintained the preload constant with time [23]. They confirm the findings of other earlier studies that have also repeatedly shown positive torque retention with titanium abutments [24]. The performance of zirconia abutions was at the middle position, whereas the post-cycling values of reverse torque were statistically lower than those of titanium but statistically higher than those of PEEK. The loss of torque seen in the group of zirconia samples (around 16 percent) is higher than the general range of values that is reported in titanium-titanium interfaces. The latter observation is consistent with the existing literature which indicates that the hardness difference between zirconia and titanium at the interface between these two materials can cause preferential wear of the softer titanium material, resulting in debris and changing the geometry of the contact [25]. This mechanism was supported in the current study through the SEM findings that concluded in the micro-chipping of the zirconia surface and wear debris. It has already been mentioned that this wear at zirconia-titanium interface may undermine the accuracy of fit and may decrease the effective clamping force with time [26]. It is worth mentioning though that the zirconia abutments in this trial included a titanium bonding base implying that the titanium-titanium screw-abutment interface was the real one and the zirconia component bonded to the titanium base. Irrespective of this design attribute, the general rigidity and stress distribution properties of the zirconia assembly is contrasted to that of a monolithic titanium abutment, which could be the cause of the intermediate performance [27]. The increased elastic modulus of zirconia over titanium may cause alternative distribution of loads in the connection, which may result in the concentration of stresses in the screw threads and an increase in the speed of preload loss [=28]. PEEK abutments showed the least maintenance of torque and the greatest amount of the torque loss (around 28 percent) and the lowest reverse torque value after cycling. This observation has clinical as well as clinical implications that can be attributed to the basic material properties of PEEK. PEEK is a semi-crystalline thermoplastic polymer which has viscoelastic behavior, i.e. it will experience time-dependent deformation when subjected to sustain loading, which is referred to as creep [29]. The material at the contact surfaces of the chewing simulator, which was PEEK under the repetitive loading conditions, was subjected to gradual plastic deformation, which was expressed as compression marks and flow of the material on the SEM examination. This distortion essentially decreases the clamping distance and subsequently it decreases the preload [30]. Moreover, since the elastic modulus of PEEK (around 3.5-4.5 Gpa) is lower than that of titanium (around 110 Gpa), the PEEK part does not transmit the applied force, but instead absorbs a higher percentage of it during elastic and plastic deformation [31]. Although this stress-absorbing effect has been suggested to be made as an advantage of decreasing stress at the bone-implant interface, the current results propose that, such effect is obtained at the cost of decreased connection stability [32]. These findings have clinical implications. A reverse torque value, which is lower than a critical value, can make the connection vulnerable to total screw loosening when further functional loading is applied. Although there is no generally agreed threshold, a minimum level of preload preservation of at least 70% of initial preload has been proposed as desirable in the long term connection stability [33]. In the current research, the titanium group retained an average of about 86% of the original preload, the zirconia group about 84% and the PEEK group about 72% implying that all the materials retained values above the critical point after the equivalent of six months of operation. Nevertheless, the much higher percentage of torque loss in the PEEK group worries about the long-term performance after the loading time studied in the present research [34]. Admittedly, the given research has some limitations that are inseparable to the in vitro type of the study. The chewing simulator offers standardisable and repeatable loading conditions but is incapable of completely simulating the complexity of the oral environment, which contains varying loading magnitudes and directions, thermal changes, pH changes and the existence of biofilm [35]. Also, the authors tested only type of implant-connection (internal hexagon) and the findings might not be applied directly to other connections in obtaining other biomechanical properties e.g., in Morse taper and external hexagon systems [36]. The lack of a crown restoration on the abutment is an additional drawback because this would change the patterns of the lever arm and stress distributions with the presence of a superstructure [37]. Future studies are advised to look into longer cycling times to reproduce overall clinical functioning, more abutment materials and types of connection and use of thermocycling as a model of thermal conditions experienced in the mouth. It requires finally clinical studies with extended follow-up to confirm the in vitro results and prove evidence-based guidelines on the choice of abutment material [38].

## Conclusion:

Abutment material significantly influences torque maintenance and screw loosening in implant-supported prostheses under simulated functional loading. Titanium abutments demonstrated superior mechanical stability with minimal torque loss, while zirconia showed intermediate performance and PEEK exhibited the highest torque loss, raising concerns about its long-term reliability. These findings highlight the importance of careful material selection, particularly in high-load regions, with emphasis on regular follow-up when using non-metallic abutments.

## Figures and Tables

**Table 1:** Baseline reverse torque values (Ncm) by abutment material group

**Group**	n	**Mean ± SD**	**Minimum**	**Maximum**	**p-value**
Titanium (Group T)	15	27.13 ± 0.98	25.4	28.6	
Zirconia (Group Z)	15	26.87 ± 1.12	24.8	28.5	0.412
PEEK (Group P)	15	26.73 ± 1.24	24.2	28.4	

**Table 2:** Post-cycling reverse torque values and percentage torque loss by abutment material group

**Group**	**n**	**Post-cycling RTV (Mean ± SD) (Ncm)**	**Torque Loss (Mean ± SD) (%)**	**p-value (within group, paired t-test)**
Titanium (Group T)	15	25.87 ± 1.42	13.77 ± 3.51	<0.001
Zirconia (Group Z)	15	22.53 ± 1.89	16.13 ± 4.22	<0.001
PEEK (Group P)	15	19.27 ± 2.14	27.87 ± 5.68	< 0.001
One-way ANOVA
revealed statistically
significant differences
among the three
groups for both
post-cycling RTV
(F = 54.83, p < 0.001)
and percentage
torque loss (F = 47.29,
p < 0.001).

**Table 3:** Post-Hoc pairwise comparisons (Tukey's HSD) for post-cycling RTV and torque loss

**Comparison**	**Mean Difference in Post-cycling RTV (Ncm)**	**p-value (RTV)**	**Mean Difference in Torque Loss (%)**	**p-value (Torque Loss)**
Titanium vs. Zirconia	3.34	0.001	-2.36	0.037
Titanium vs. PEEK	6.6	< 0.001	-14.1	< 0.001
Zirconia vs. PEEK	3.26	0.001	-11.74	< 0.001
